# Replacing the Concentrate Feed Mixture with *Moringa oleifera* Leaves Silage and *Chlorella vulgaris* Microalgae Mixture in Diets of Damascus Goats: Lactation Performance, Nutrient Utilization, and Ruminal Fermentation

**DOI:** 10.3390/ani12121589

**Published:** 2022-06-20

**Authors:** Ahmed E. Kholif, Gouda A. Gouda, Amgad A. Abu Elella, Amlan K. Patra

**Affiliations:** 1 Dairy Science Department, National Research Centre, 33 Bohouth St. Dokki, Giza 12622, Egypt; gagouda@gmail.com; 2 Animal Production Research Institute, Agriculture Research Center, Dokki, Giza 12126, Egypt; amgadahmed1959@gmail.com; 3 Department of Animal Nutrition, West Bengal University of Animal and Fishery Sciences, 37 K.B. Sarani, Kolkata 700037, India; patra_amlan@yahoo.com

**Keywords:** associative effects, foliage trees, milk production, microalgae, ruminal fermentation

## Abstract

**Simple Summary:**

*Moringa oleifera* and *Chlorella vulgaris* microalgae have a good balance of amino acids with high protein contents; however, complementary and synergic effects between proteins can improve their nutritive value compared to the individual additive. Replacing a concentrate mixture at 20% to 40% levels with a mixture of *M. oleifera* and microalgae improved nutrient digestibility, ruminal fermentation characteristics, milk production, composition and the fatty acid profile of goats. Inclusion of *M. oleifera* and microalgae mixture to partially replace concentrates may enhance milk production performance and milk quality without affecting animal health.

**Abstract:**

Exploring suitable alternatives for high-cost concentrate feeds is a critical factor for successful livestock production. The present experiment aimed to evaluate the dietary inclusion of *Moringa oleifera* silage and *Chlorella vulgaris* microalgae (at 1% of total diet, DM basis) in a quintuplicate 3 × 3 Latin square design for milk production performance, nutrient utilization and ruminal fermentation in Damascus goats. Fifteen lactating Damascus goats were divided into three groups to be fed a diet composed of a concentrate mixture and rice straw at 60:40 (DM basis) in the control group and fed for 30 days in each period. The concentrate mixture in the control treatment was replaced with *M. oleifera* silage and *C. vulgaris* microalgae at 20% (MA20 treatment) or 40% (MA40 treatment). Treatments did not affect total feed intake but increased (*p* < 0.01) crude protein (CP) and fiber intakes while decreasing nonstructural carbohydrates intake. The digestibility of CP and acid detergent fiber increased due to silage supplementation compared to the control treatment. Treatments increased (*p* < 0.05) ruminal pH and the concentrations of total volatile fatty acids, acetate, and propionate; however, they decreased (*p* < 0.05) the concentrations of ammonia-N. Treatments increased (*p* < 0.05) the concentrations of serum glucose and antioxidant capacity. Both MA20 and MA40 treatments increased the daily milk production, the concentrations of milk fat and lactose, and feed efficiency compared to the control treatment. Additionally, MA20 and MA40 treatments increased the proportions of total polyunsaturated fatty acids and total conjugated linoleic acids. It is concluded that the concentrate feed mixture in the diet of lactating goats can be replaced up to 40% (equals to 24% of total diet) with *M. oleifera* silage to improve feed intake and nutrient utilization, and milk production performance.

## 1. Introduction

One of the main challenges faced by animal nutritionists is the scarcity and high cost of concentrates, which forces scientists to explore suitable alternatives for ruminant and nonruminant animals [1,2]. Use of multipurpose tree leaves (e.g., *Moringa oleifera*) and protein-rich microalgae (e.g., *Chlorella vulgaris*) in animal feeding has gained increasing interest, with mixed results [3,4,5,6]. Usually, supplementation of foliages in low-quality forage diets at low levels (20 to 40% of the total diet) is beneficial for ruminant performance and nutrient utilization due to better ruminal microbial activities. The multipurpose tree leaves are important low-cost feed resources for smallholder farmers in the low-income countries.

*M. oleifera* is a rapid-growing softwood tree that grows in all tropical and subtropical areas and can adapt to different environmental and soil conditions [2], making it available throughout the year. The proximate analysis revealed that *M. oleifera* leaves contain crude protein (CP) content (23.0–29.4%), fat (4.03–9.51%), mainly α-linolenic acid, fiber (6.00–9.60%), and ash (8.05–10.38%). Additionally, *M. oleifera* leaves contain vitamin C (188–279 mg/100 g), Ca (1.32–2.65%), P (0.152–0.304 g/100 g), and K (1.32–2.03 g/100 g) [2,7]. Moreover, protein in the *M. oleifera* leaves has about 47% rumen bypass protein [8] with a good amino acid profile [9]. A recent experiment partially replaced protein feeds (e.g., soybean and sesame meals) in the diets of ruminants with unconventional alternatives (e.g., plant leaves rich in protein) and observed increased nutrient intake and digestibility and altered ruminal fermentation (e.g., increased concentrations of ruminal acetic, propionic, and total volatile fatty acids), and improved final body weight, and daily weight gain [10]. Replacement of berseem clover with *M. oleifera* leaves in the diets of lactating goats improved feed efficiency and milk production [6].

*C. vulgaris* is a fresh-water, unicellular microalga, with a high concentration of CP (about 600 g CP/kg DM) containing all essential amino acids [11,12]. The main amino acids in *C. vulgaris* are glutamic acid and leucine with relatively high concentrations of lysine and methionine, which are the first two limiting amino acids in animal nutrition [2]. *C. vulgaris* also contains other biological active components such as antioxidants, provitamins, vitamins, pigments, a growth phytonutrient known as the *C. vulgaris* growth factor (CGF), unsaturated fatty acids (UFA), glycoproteins and carotenoids [11]. Experiments [3,13] showed that *C. vulgaris* improved ruminal bacterial growth and altered ruminal biohydrogenation of monounsaturated (MUFA) and polyunsaturated fatty acids (PUFA) due to their effects on ruminal microbes, especially those involved in the metabolism and biohydrogenation of fatty acids in the rumen [11,14]. Recently, Kholif et al. [3] observed that the inclusion of *C. vulgaris* in the diets of lactating Boer goats improved nutrient intake and digestibility, ruminal fermentation, lactational performance and milk nutritive value; however, other researchers [15] observed weak effects on feed intake, digestibility or daily milk production when feeding *C. vulgaris* to lactating Finnish Ayrshire cows.

In an in vitro study, we evaluated three levels (1%, 2% and 3%) of *C. vulgaris* and twelve levels (0 to 100%) of *M. oleifera* silage to decide optimum levels of these two ingredients in diets and we noted that 1% of *C. vulgaris* and up to 40% of *M. oleifera* in the diets were the best to improve ruminal fermentation. However, there is limited information on the synergic effects of *C. vulgaris* microalgae and *M. oleifera* as concentrate feeds on feed utilization and lactation performance of lactating goats. Based on the literature on *C. vulgaris* and *M. oleifera* as feed ingredients in ruminant diets, we hypothesized that a combination of these two ingredients could enhance milk production performance in goats. Accordingly, this experiment aimed to evaluate two replacement levels of concentrates with *M. oleifera* leaves silage in the presence of *C. vulgaris* microalgae at 1% of total diet (DM basis) on nutrient utilization, ruminal fermentation, biochemical blood parameters and milk production in lactating Damascus goats.

## 2. Materials and Methods

### 2.1. Study Location

This study was carried out in the experimental farm at Gemmeiza Station of the Animal Production Research Institute, Egypt. Management of the does was in accordance with the 3rd edition (2010) of the guide of Agricultural Research and Teaching of the Federation of Animal Science Societies, Champaign, IL, USA and approved by the Institutional Animal Care and Use Committee of the Animal Production Research Institute, Egypt.

### 2.2. Moringa Oleifera and Chlorella Vulgaris Microalgae Cultivation

*M. oleifera* seeds were planted at a density of 100,000–150,000 seeds per ha. The field was irrigated with 900 m^3^ water/ha biweekly without any fertilizer. When plant height reached to 65–70 cm, a first uniformity cutting was carried out at 5–7 cm cutting height 65 days after seeding. This cut was used for feeding other animals, not for the animals used in the present experiment. A second cut of *M. oleifera* (45 days after the first cut) biomass, composed of leaves and small twigs was harvested and large twigs were removed. Usually, *M. oleifera* results nine harvests per year and yielding 70–80 tons of fresh biomass/ha/year (∼23 tons DM/ha/year). The material (about 1 ton) was left on the field for 1 h and then chopped and used to prepare silage used in the present experiment. Molasses was mixed at 5% of fresh weight. About 40 kg fresh materials per bag was packed into a polythene silo bag (40 × 70 cm) and compressed manually for quick creation of anaerobic conditions. The bags were sealed and stored indoors on a dry concrete floor for 45 days. Samples of ensiled materials were collected from five different bags (1 kg/bag), dried and kept for silage evaluation and chemical analysis. Silage pH, ammonia-N (NH_3_-N) and volatile fatty acids (VFA) were analyzed as quality indicators of silage according to AOAC [16]. Aflatoxin F_1_ concentration was determined in silage with the use of a Fluorometer, Series-4 (Vicam, Milford, MA, USA) based on the method described by AOAC [16]. Tannin [17] and total phenolic concentration [18] in *M. oleifera* silage were determined following standard protocols.

Laboratory production of *C. vulgaris* was performed using 5 L glass flasks containing 3 L algal growth medium. Pure strain of *C. vulgaris* H1957 was obtained from the Marine Toxins laboratory, National Research Centre, Egypt. The culture media for cultivation of *C. vulgaris* was BG-11 medium containing (/L) 1.5 g NaNO_3_, 0.04 g K_2_HPO_4_, 0.075 g MgSO_4_.7H_2_O, 0.036 g CaCl_2_.2H_2_O, 0.006 g citric acid, 0.006 g ferric ammonium citrate, 0.001 g EDTA, 0.02 g Na_2_CO_3_, and 1 mL trace-metal mix A5 [19]. After autoclaving and cooling, the pH of the medium was adjusted to 7.1. *C. vulgaris* was cultivated under continuous illumination with white fluorescent lamps at room temperature and aeration was performed using an air compressor linked with polyethylene tubes (3 mm). After 25 days, *C. vulgaris* in their late exponential phase was transferred at 1:10 into 1000 L polyethylene tanks (*n* = 5) containing 600 L culture media and linked with an aeration system. *C. vulgaris* biomass was harvested using the continuous separating centrifuge apparatus (Westevalia Separator centrifuge at 15,000 L/h) and drained water was recycled to the ponds. The harvested biomass (0.75 kg microalgae per day) was re-washed three times with tap water to remove any residues of salts from the culture media. Biomass was then partially dried using an air-drying oven at 45 °C for 2–4 h.

### 2.3. Goats, Feeding and Management

Fifteen lactating Damascus does (mean ± SD: 2 ± 0.5 parity, 41.0 ± 1.5 kg body weight, 24 ± 4.1 months of age, 850 ± 30.5 g/d of previous milk production, twin birth, normal suckling) in the first week of lactation were randomly assigned to three experimental treatments in a quintuplicate 3 × 3 Latin square design. The experimental design had three treatments, three periods and five does per treatment within each period, resulting in 15 replicates per treatment. The three experimental treatments were assigned randomly to the three groups in the first period, after which a predetermined sequence was followed that allowed each doe to receive each treatment.

Does were individually housed in semi-opened concrete floor pens (1.5 m^2^/goat) under shade, without bedding and with free access to water. Kids were kept with their mothers throughout the experimental period, with the exception of days when feed intake and nutrient digestibility were determined. Does were offered the experimental diets to meet their minimum CP and net energy requirements according to NRC [20] recommendations plus 10% extra allowance.

The basal diet fed to the goats (control treatment) contained rice straw and a concentrate feed mixture at 40:60 (DM basis). In the other experimental diets, a mixture of *M. oleifera* silage and *C. vulgaris* microalgae (at 10 g/kg DM), produced as previously mentioned, replaced the concentrate mixture at 20% (MA20 treatment) or 40% (MA40 treatment) on DM basis. The replacement levels were recommended by an in vitro experiment (unpublished data). Does were offered the allotted amounts of concentrate feed mixture mixed with *C. vulgaris*, followed by *M. oleifera* silage and then rice straw. The ingredients and chemical composition of the diets are presented in Table 1.

### 2.4. Feed Intake and Apparent Nutrient Digestibility

Diets were offered to the does individually at 08:00 and 16:00 h in two equal amounts. Each experimental period lasted 30 days: 20 days of adaptation to the new diet, and 10 days for measurements (feed intake and milk yield) and sample collection (sampling of feed and orts, feces, ruminal fluid, blood and milk). Three digestibility trials were conducted during the last 10 d of each experimental period (d 20–30, d 50–60 and d 80–90) to determine apparent total tract nutrient digestibility by a marker method. In each day, the offered feeds and orts amounts were recorded individually for each goat. Daily orts of individual feeds (concentrate feed mixture mixed with *C. vulgaris*, *M. oleifera* silage and rice straw) from the two times of feeding were individually collected and pooled for each doe before sampling. During sample collection periods, daily feed intake was measured as the difference between feed offered and orts from the previous day’s feeding. During collection periods, individual fecal samples from all does were collected twice daily at 07:00 and 15:00 h, dried at 60 °C in a forced-air oven for 48 h, and pooled per doe. Nutrient intake was calculated by multiplying the total intake by nutrient concentration in the feed. Acid-insoluble ash was used as an internal indigestibility marker, and coefficients of digestion were calculated according to Ferret et al. [23]. Goats were weighed monthly on a digital multi-purpose platform scale. Diets were sampled daily, composited weekly, dried at 60 °C in a forced-air oven for 48 h [16] (method 930.15), and stored pending chemical analyses.

Composited samples of dried feeds, orts of each feed and feces were ground to pass through a 1-mm screen using a Wiley mill, and analyzed for different components (nitrogen, ether extract and ash) according to AOAC [16] official methods. Neutral detergent fiber (NDF) content was determined according to Van Soest et al. [24] with use of alpha amylase and sodium sulfite and expressed without residual ash. Acid detergent fiber (ADF) [16] and lignin [24] contents were determined. Concentrations of non-structural carbohydrates (NSC), cellulose, hemicellulose, and organic matter (OM = 1000–ash) were calculated. Total digestible nutrient and energy content of diets were estimated according to NRC [21] and INRA [22] equations.

### 2.5. Sampling and Analysis of Rumen Fluid

On the last day of each experimental period, ruminal contents were sampled 3 h after the morning feeding to determine the pH and concentration of fermentation end-products. After initial discarding of 50 mL ruminal fluid, 100 mL ruminal fluid were collected by using a stomach tube, and the samples taken from each doe were strained through four layers of cheesecloth. Ruminal fluid pH was measured immediately using a pH meter (HI98127 pHep^®^4 pH/Temperature Tester, Hanna^®^ Instruments, Villafranca Padovana PD, Italy).

A subsample of 5 mL ruminal fluid was preserved with 5 mL of 0.2 *M* HCl for ammonia-N analysis [16], and 0.8 mL of ruminal fluid was mixed with 0.2 mL of a solution containing 250 g of metaphosphoric acid/L for total volatile fatty acids (VFA) analysis. Samples were preserved at −20 °C pending analyses. Concentration of VFA and its individual molar proportions were determined using a gas chromatograph (Thermo Fisher scientific, Inc., TRACE1300, Rodano, Milan, Italy) fitted with an AS3800 autosampler and equipped with a capillary column HP-FFAP (19091F-112; 0.320 mm o.d., 0.50 μm i.d., and 25 m length; J & W Agilent Technologies Inc., Palo Alto, CA, USA). A mixture of known concentrations of individual short-chain fatty acids (acetate, propionate and butyrate) was used as an external standard (Sigma Chemie GmbH, Steinheim, Germany) to calibrate the integrator.

### 2.6. Sampling and Analysis of Blood Serum

On the last day of each experimental period, blood samples (10 mL) were collected 4 h after feeding from the jugular vein of each doe into a clean dry tube without anticoagulants. Blood samples were centrifuged at 4000× *g* at 4 °C for 20 min. Serum was separated into 2-mL clean dried Eppendorf tubes and frozen at −20 °C until analysis. Concentrations of blood parameters were enzymatically analyzed in blood serum samples using specific kits (Stanbio Laboratory, Boerne, Texas, USA), following manufacturer instructions.

### 2.7. Milk Sampling and Composition

During the last 10 days of each experimental period, does were milked by hand twice daily at 09:00 and 21:00 h, amount of milk yield was measured in a weighing balance, and milk samples (10% of recorded milk yield) were collected at each milking. A mixed sample of morning and evening milk was taken daily. Milk samples were analyzed for different components using infrared spectrophotometry (Lactostar Dairy Analyzer, Funke Gerber, Berlin, Germany).

Fatty acid contents in milk were determined in fatty acid methyl esters prepared by base-catalyzed methanolysis of the glycerides (potassium hydroxide in methanol) according to international standards (ISO 15884-IDF 182. 2002, Brussels, Belgium: International Dairy Federation) on a Perkin-Elmer chromatograph (model 8420, Beaconsfield, Perkin Elmer, Beaconsfield, UK) equipped with a Cp-Sil 88 fused-silica capillary column (100 m length × 0.25 mm internal diameter × 0.2 µm film thickness; Chrompack, Middelburg, Netherlands) and a flame ionization detector (HP, Little Falls, DE, USA). The atherogenic index (AI) was calculated according to Ulbricht and Southgate [25].

Average yield (g/d) of each milk component was calculated by multiplying milk yield by the component content (g/kg). Gross energy content in milk was calculated according to Tyrrell and Reid [26]. Milk energy output (MJ/d) was calculated as milk energy (MJ/kg) × milk yield (kg/d). Fat-corrected milk (FCM, kg/day) and energy-corrected milk (ECM, kg/day) were calculated according to Tyrrell and Reid [26]. Feed efficiency was calculated and expressed as milk yield, FCM, and ECM per unit of DM intake. Feed efficiency was calculated as milk: intake, ECM: intake and FCM: intake ratios.

### 2.8. Statistical Analyses

The Shapiro–Wilk test was used to test the normal distribution of data. For the small number of variables that showed significance for the Shapiro–Wilk test, data transformation (e.g., natural log, inverse of the natural log, square root, or inverse of the square root) was applied before statistical analysis. Data were analyzed using a quintuplicate 3 × 3 Latin square design, with three periods and three treatments. The statistical model included the fixed effect of square and treatment, and the random effects of period and goat nested within square: Y_ijkl_ = μ + S_i_ + T_j_ + P_k_ + G_l_(S_i_) + E_ijkl_, where Y_ijkl_ is each individual observation for a given variable, μ is the overall mean, S_i_ is the square effect, T_j_ is the treatment effect, P_k_ is the period effect, G_l_(S_i_) is the effect of goat within square and E_ijkl_ is the residual error. Statistical analyses were performed using PROC MIXED of SAS (Online Version, SAS^®^ OnDemand for Academics, SAS Inst. Inc. Cary, NC, USA). When the treatment *F*-test was significant at *p* < 0.05, means were then compared by applying the probability of difference option of the least squares means statement. The contrast between control versus silage treatments was used to test for differences between control diet versus both *M. oleifera* leaves silage and *C. vulgaris* microalgae diets. Significance was declared at a level of *p* < 0.05.

## 3. Results

### 3.1. Feed Intake and Apparent Nutrient Digestibility

Treatments did not affect the intakes of DM, organic matter (OM) and net energy for lactation; however, increased crude protein (CP, *p* < 0.001) intakes and decreased (*p* < 0.001) NSC intake were observed with increasing levels of silage in diets (Table 2).

Increased (*p* < 0.01) digestibility of DM, OM, CP, ether extract (*p* < 0.05), NDF and ADF were observed with increasing levels of dietary *M. oleifera* silage and *C. vulgaris* microalgae (Table 2).

### 3.2. Ruminal Fermentation

Ruminal pH (*p* < 0.01) and the concentrations of total VFA, acetate and propionate (*p* < 0.05) were increased with feeding MA20 and MA40 diets, while both silage diets decreased (*p* = 0.018) the concentrations of ammonia-N (Table 3). Treatments did not affect the concentrations of butyrate and acetate: propionate ratio.

### 3.3. Blood Chemistry

The serum concentrations of globulin, urea-N, alanine aminotransferase (ALT), aspartate amino transferase (AST), triglycerides, low density lipoprotein (LDL), beta-hydroxybutyric acid (BHBA) and non-esterified fatty acids (NEFA) were not affected by diets in the serum of lactating goats (Table 4). Increasing levels of silage in the diets increased (*p* < 0.05) serum glucose, triglycerides, high-density lipoprotein (HDL), and antioxidant capacity.

### 3.4. Milk Yield, Composition, and Fatty Acids

Experimental diets increased (*p* < 0.01) the daily production of milk, ECM, FCM and yields of milk components (Table 5). Additionally, diets containing silage increased (*p* < 0.01) the concentrations of milk fat, lactose, and milk energy compared to the control diet. Moreover, the MA20 and MA40 treatments increased (*p* < 0.01) feed efficiency calculated as milk: intake, ECM: intake or FCM: intake ratio compared to the control treatment.

### 3.5. Milk Fatty Acids

Treatments affected some of the individual fatty acids in milk (Table 6). Increases (*p* < 0.05) in the proportions of C8:0, C20:5n3 and C22:5n3 were observed with the experimental diets, while increases (*p* < 0.05) in the proportions of C15:0 and C16:1 fatty acids were observed with increasing levels of dietary silage. Also, increases (*p* < 0.01) in the proportions of C18:1n9 *cis*, C18:1n9 *trans*, C18:2 *trans*-10, *cis*-12, C18:2 *cis*-9, *trans*-11, C18:3n6, C20:5n3, C22:5n3, C20:0, polyunsaturated fatty acids (PUFA) and total conjugated linoleic acids (CLA) as well as the unsaturated fatty acids to saturated fatty acids (UFA: SFA) ratio were noted in the silage diets. However, a decreased (*p* < 0.01) proportions of C16:0 and the atherogenicity index were observed with increasing levels of silage in the diets.

## 4. Discussion

The objective of this study was to evaluate the moringa silage replacing 20 to 40% standard concentrate mixture in the diet of lactating goats. The moringa silage (282 g/kg DM) contained a greater amount of CP compared with the standard concentrate mixture (162 g/kg DM). The diet was not formulated to have similar CP and net energy content to avoid the confounding effect of ingredient composition that could mask the actual effect of moringa silage. Therefore, the diets containing moringa silage had higher concentration of CP and net energy that might contribute to the production performance, in addition to other factors.

### 4.1. Feed Intake and Nutrient Apparent Digestibility

Without affecting DM or NDF intake, the MA20 and MA40 treatments increased the intakes of CP (by 14.1 and 26.7%, respectively), while decreasing NSC intake (by 10.2 and 18.4%, respectively). This is a net result of the different concentrations of CP, fiber and NSC in *M. oleifera*, *C. vulgaris* and concentrates. No effect on DM intake of feeding *M. oleifera* [27] or *C. vulgaris* [3,15] in diets of lactating goats were reported earlier. Others reported that *M. oleifera* has a high palatability and feeding it to animals increased feed intake [6].

The MA20 and MA40 treatments increased the digestibility of DM (by 9.8 and 11.0%, respectively), OM (by 11.1 and 13.8%, respectively), CP (by 13.7 and 14.2%, respectively), NDF (by 8.3 and 11.9%, respectively) and ADF (by 7.5 and 10.6%, respectively) compared with the control, which may be related to improved ruminal fermentation and microbial activity with the experimental diets. *C. vulgaris* is reported to contain CGF and *β*-glucan, which can scavenge free radicals, resulting in improved digestion and ruminal fermentation [11]. Moreover, *C. vulgaris* increased the relative proportions of ruminal *Butyrivibrio fibrisolvens*, *Ruminococcus albus* and *Clostridium sticklandii* in goats and increased ruminal digestibility [28,29]. The presence of carotenoids, phycobiliproteins, polysaccharides and phycotoxins in *C. vulgaris,* which can stimulate microbial growth, is another reason for improving digestibility [11,30]. *M. oleifera* contains a good portion of secondary metabolites [31], which may also be responsible for the improved nutrient digestibility. Recently, Ebeid et al. [31] stated that *M. oleifera* contains secondary metabolites that increase the number and diversity of ruminal microbes responsible for nutrient fermentation. Moreover, the increased CP intake may be considered as another factor for the improved apparent digestibility because the proportion of metabolic fecal nitrogen decreases with the increase of CP intake. Also, the differences in NDF digestibility of the ingredients of the concentrates and *M. oleifera* may be responsible improved NDF digestibility of the *M. oleifera* diets. Increased total tract digestibility of CP with lowered ruminal ammonia-N concentration confirms the beneficial effect of the tannins in *M. oleifera*, which form a tannin–protein complex in the rumen and escape into the small intestine causing increased protein digestion in the lower gut [32].

### 4.2. Ruminal Fermentation

The observed increases in ruminal pH by 8.2% and 8.9% for MA20 and MA40 treatments, respectively, may be related to the increased concentration of fiber in the diets as a result of replacing the concentrate mixture (low fiber concentration) with *M. oleifera* (high fiber concentration) silage. Increasing ruminal pH is an important factor for increasing nutrient, especially fiber, digestion [33].

Treatments MA20 and MA40 increased the concentrations of total VFA by 9.1 and 11.6%, respectively, and propionate (by 9.1% and 11.6%, respectively), which may be a result of increased nutrient digestibility [3,6]. Increasing the concentrations of total VFA and decreasing ammonia-N at the same time are nutritionally desirable for enhancing production performance of ruminants [34]. Increasing the concentration of ruminal propionate is also advantageous in lactating animals because milk lactose synthesis depends upon propionate [35]. Feeding *M. oleifera* [6] and *C. vulgaris* microalgae [3] increased the concentrations of total VFA and propionate in the rumen of lactating goats.

MA20 and MA40 treatments increased the concentrations of acetate by 10.7% and 11.7%, respectively, as a result of increased fiber digestion. Feeding of *C. vulgaris* [3,36] and *M. oleifera* [6] also increased the concentrations of ruminal acetate in earlier studies. Increasing fiber intake and digestion favor the growth of acetate-producing rumen bacteria. Greater ruminal acetate concentration can increase milk fat content, as discussed later. The concentrations of ammonia-N decreased (9.7% and 10.2% for MA20 and MA40 treatments, respectively) in the rumen, which is inconsistent with the observed increased CP digestion. Results of ruminal ammonia-N were expected since both of *C. vulgaris* and *M. oleifera* contain a ruminal low-degradable protein [9,11]. As previously noted, the presence of tannins in *M. oleifera*, nucleic acids, nitrogen-containing cell walls, and amines in *C. vulgaris* [37] would also be the reasons for low concentrations of ruminal ammonia-N. Moreover, *M. oleifera* supplementation reduces urease activity and lowers the number of protozoa in the rumen [10].

### 4.3. Blood Chemistry Measurements

In the present experiment, all measured parameters were within the ranges reported for healthy animals [38]. The negligible effects of diets on serum total protein, albumin, globulin or urea-N indicate normal kidney function [39]. Additionally, treatments did not affect the concentrations of ALT and AST, suggesting unaffected liver health and function and absence of hepatotoxicity with feeding treatments. Other reported similar results with feeding of *C. vulgaris* microalgae [11] and *M. oleifera* [6] to lactating goats. Both MA20 and MA40 treatments did not affect the concentrations of serum BHBA or NEFA, indicating that body-fat mobilization and net energy balance were not differed among the groups [40].

The MA20 and MA40 treatments increased triglycerides (by about 4.9%) and good cholesterol (i.e., HDL) by 12.0% and 13.1%, respectively, indicating that a mixture of *C. vulgaris* microalgae and *M. oleifera* silage can be used as an approach for improving the animals’ lipid profile. The present results may be related to the presence of phenolic compounds in the leaves of *M. oleifera*, and the hypocholesterolemic actions of *C. vulgaris* microalgae [11]. Microalgae are able to modify the lipoprotein metabolism and alter plasma lipid profile due to their α-glucan [41]. The increased serum glucose (by 10.5% and 10.9% for MA20 and MA40 treatments, respectively) may be related to the increased OM digestibility, increased ruminal total VFA and propionate concentrations [35].

The MA20 and MA40 treatments increased serum antioxidant capacity by 9.5% and 11.5%, respectively, which might be related to the presence of polyphenols and antioxidants such as isothiocyanates in *M. oleifera* [42] and *C. vulgaris* [43]. Cohen-Zinder et al. [42] observed that feeding *M. oleifera* silage increased the concentration of antioxidants in lactating cows due to the presence of isothiocyanates and accumulation of amino acids and low-molecular-weight peptides.

### 4.4. Milk Yield and Composition

Silage and *C. vulgaris* feeding (MA20 and MA40 treatments) increased the daily milk production (14.5% and 16.8%, respectively), ECM (27.1% and 22.8%, respectively) and FCM (23.9% and 20.5%, respectively). Moreover, higher milk production with no effect on feed intake in the experimental diets indicates an enhanced feed efficiency (milk: intake ratio by 17.6% and 12.6%, ECM: intake ratio by 28.2% and 25.4%, FCM: intake ratio by 25.7% and 22.9% for MA20 and MA40 treatments, respectively). The increased milk production may be due to the accumulative effects of the different chemical composition of the diets, mainly CP, greater nutrient digestibility, ruminal total VFA and propionate and serum glucose concentrations. Many experiments showed positive effects of *M. oleifera* silage [42] and *C. vulgaris* [3,15] on milk production.

*M. oleifera* contains a considerable concentration of ruminal undegraded protein (about 47%), secondary metabolites, and several essential nutrients (such as amino acids, essential fatty acids, vitamins and minerals), which may increase milk production [6,9]. Calculated feed efficiency relative to CP intake was lower for M20 and M40 than for control goats (6.48 vs. 6.51 and 5.98 kg of milk/kg CP intake, respectively), suggesting that CP intake was probably one of the main factors influencing milk yield. As previously noted, treatments increased ruminal propionate concentration, which can increase lactose synthesis and consequently milk yield [35]. This effect may also explain the observed increase concentration of lactose in milk by 9.4% and 6.3% for MA20 and MA40 treatments, respectively.

Treatments increased the concentrations of milk fat by 10.7% and 9.4% for MA20 and MA40 treatments, respectively, which is consistent with an observed increase in fiber digestion and ruminal acetate concentrations in goats fed the experimental diets. Milk fat can be synthesized from acetate, leading to greater amounts of milk fat precursors in blood [3,15]. Similar results were observed in other experiments with *C. vulgaris* [15] or *M. oleifera* feeding [6]. Lamminen et al. [15] observed that feeding *C. vulgaris* to lactating animals increased ruminal acetate and enhanced mammary uptake of acetic acid about twofold.

### 4.5. Milk Fatty Acids

It is well documented that milk fatty acids profile is highly sensitive to dietary changes [14,36]. Concentrations of milk fatty acids are greatly influenced by the type of fatty acids consumed by animals. Feeding a mixture of *M. oleifera* and *C. vulgaris* decreased the atherogenicity index by 14.1% and 26.7%, respectively, and increased the proportions of total PUFA by 9.8% and 9.8%, respectively, and of CLA by 11.1% and 11.1%, respectively. The ratio of UFA: SFA by 7.0% and 9.3% for MA20 and MA40 treatments, respectively. Such milk fatty acid profiles are healthier for consumers. In a review, Altomonte et al. [44] summarized that the greatest changes in milk fatty acid profile were associated with increases in long-chain PUFA and n-3 fatty acids accompanied by decreased concentration of SFA. A high portion of fatty acids in milk is transferred directly from feeds after intestinal absorption. Therefore, the different profiles of fatty acids in the concentrate mixture and the *M. oleifera* and *C. vulgaris* mixture, respectively, may partially explain the observed results. In the present experiment, the increased concentration of C18 fatty acids in milk may be related to increased mammary supply of C18:0 with *M. oleifera* and *C. vulgaris* feeding, providing more substrate for mammary Δ^9^-desaturase [45].

Most dietary PUFA undergo extensive ruminal biohydrogenation and thus the fatty acid profile in milk is highly different from the dietary fatty acid profile. Protecting PUFA from ruminal biohydrogenation can increase their secretion in milk. Moreover, *M. oleifera* silage contains polyphenolic compounds (49 g/kg DM), including tannins (19 g/kg DM), that might also be responsible for reduced biohydrogenation of PUFA in the rumen [46] and consequently greater PUFA and CLA content in milk and meat [46]. Nonetheless, *de novo* desaturation of fatty acids also determines the PUFA and CLA content in milk. Feeding the mixture of *M. oleifera* and *C. vulgaris* might alter the ruminal microbiota, causing changes in the structure of fatty acids from the diet [14,47]. Feeding UFA-rich feeds such as *M. oleifera* [9] and *C. vulgaris* [3] to lactating animals also altered fatty acid profile (i.e., increased UFA and CLA proportion and decreased SFA proportions) in milk.

## 5. Conclusions

Replacement of a concentrate feed mixture with a mixture of *M. oleifera* and *C. vulgaris* improved nutrient digestion, ruminal fermentation characteristics, milk yield, and milk composition; increased relative proportions of unsaturated fatty acids and conjugated linoleic acids in milk; and decreased proportions of SFA. The recommended level of replacement of was up to 40% (equal to 24% of total diet DM) under the current experimental conditions. The CP content in the *M. oleifera* diets was greater than the control diet, which might result in improved milk production performance, in addition to other nutraceutical factors of the *M. oleifera* silage. Further studies are warranted to study the effect of feeding the *M. oleifera* and *C. vulgaris* mixture with similar CP content on rumen microbiota composition, to understand the mechanisms of improved nutrient digestibility and ruminal fermentation.

## Figures and Tables

**Table 1:** Ingredients (g/kg DM) and chemical composition (g/kg DM) of total mixed rations fed to the lactating Damascus goats.

	Ingredient				Diet ^1^		
	Rice Straw	Concentrate Feed Mixture ^2^	*Moringa oleifera* Silage ^3^	*Chlorella vulgaris* Microalgae	Control	MA20	MA40
Ingredient							
Rice straw					400	400	400
Concentrate feed mixture					600	480	360
*Moringa oleifera* silage					0	110	230
*Chlorella vulgaris* microalgae					0	10	10
Chemical composition							
Dry matter	943	838	391	932	880	832	778
Organic matter	849	891	862	942	874	872	868
Crude protein	43	162	282	579	114	132	146
Ether extract	19	42	45	139	33	34	34
Non-structural carbohydrates	159	421	190	106	316	288	260
Neutral detergent fiber	628	266	345	118	411	418	427
Acid detergent fiber	397	99	299	43	218	240	264
TDN (g/kg DM) ^4^					507	540	545
DE (Mcal/kg DM) ^4^					2.24	2.38	2.40
ME (Mcal/kg DM) ^4^					2.26	2.41	2.43
NEL (Mcal/kg DM) ^4^					1.12	1.20	1.21
UFL (Mcal/kg DM) ^5^					1.98	2.12	2.14

^1^ Diets: Concentrate mixture in the control diet was replaced with *Chlorella vulgaris* microalgae (at 1%) and *Moringa oleifera* silage mixture at 0% (Control diet), 20% (MA20 diet) or 40% (MA40 diet), DM basis. ^2^ Contained per kg DM: 250 g un-decorticated cotton seed meal, 350 g wheat bran, 300 g maize, 30 g rice bran, 30 g molasses, 20 g limestone, 10 g urea and 10 g salt. ^3^ *M. oleifera* silage measurements: pH = 4.2, ammonia-N = 51 g/kg of total N, volatile fatty acids = 88 g/kg DM, aflatoxin F_1_ = 1.1 µg/kg of DM, total phenolics = 49 g/kg DM, and tannins = 19 g/kg DM. ^4^ TDN = total digestible nutrients, DE = Digestible energy, ME = Metabolizable energy, NEL = Net energy for lactation. All have been calculated according to NRC [21] equation. ^5^ UFL = unité fourragère du lait (net energy requirements for lactation equivalent of 1 kg of standard air-dry barley) calculated according to INRA [22] equation.

**Table 2:** Nutrient intake and digestibility of diets containing *Moringa oleifera* and *Chlorella vulgaris* microalgae fed to lactating Damascus goats (*n* = 15).

	Diet ^1^				*p* Values	
	Control	MA20	MA40	SEM	Diet	Control vs. others
Intake (g/d)						
Dry matter	1181	1167	1172	7.4	0.401	0.200
Organic matter	1033	1017	1017	6.5	0.160	0.057
Crude protein	135 ^c^	154 ^b^	171 ^a^	0.9	<0.001	<0.001
Non-structural carbohydrates	374 ^a^	336 ^b^	305 ^c^	2.2	<0.001	<0.001
Neutral detergent fiber	485	488	501	6.1	0.290	0.221
Net energy for lactation (Mcal/d)	1.32	1.40	1.42	0.147	0.063	0.052
Digestibility (g digested/kg ingested)						
Dry matter	553 ^b^	607 ^a^	614 ^a^	7.9	<0.001	<0.001
Organic matter	559 ^b^	621 ^a^	636 ^a^	7.5	<0.001	<0.001
Crude protein	549 ^b^	624 ^a^	627 ^a^	6.4	<0.001	<0.001
Ether extract	582 ^b^	623 ^a^	627 ^a^	7.7	0.002	<0.001
Non-structural carbohydrates	591	607	597	9.8	0.509	0.379
Neutral detergent fiber	521 ^b^	564 ^a^	583 ^a^	9.2	<0.001	<0.001
Acid detergent fiber	517 ^b^	556 ^a^	572 ^a^	8.0	<0.001	<0.001

^a,b,c^ Means in the same row with different superscripts differ at *p* < 0.05. *p*-value is the observed significance level of the *F*-test for treatment; SEM, standard error of the mean. ^1^ Diets: Concentrate mixture in the control diet was replaced with *Chlorella vulgaris* microalgae (at 1%) and *Moringa oleifera* silage at 0% (Control diet), 20% (MA20 diet) or 40% (MA40 diet), DM basis.

**Table 3:** Ruminal fermentation parameters of lactating Damascus goats fed diets containing *Moringa oleifera* and *Chlorella vulgaris* microalgae (*n* = 15).

	Diet ^1^				*p* Values	
	Control	MA20	MA40	SEM	Diet	Control vs. Others
pH	5.59 ^b^	6.05 ^a^	6.09 ^a^	0.053	<0.001	<0.001
Ammonia-N, mg/dL	32.2 ^a^	29.8 ^b^	28.9 ^b^	0.57	0.018	0.006
Total volatile fatty acids, mmol/L	121 ^b^	132 ^a^	135 ^a^	3.1	0.008	0.002
Acetate, mmol/L	72.6 ^b^	80.4 ^a^	81.1 ^a^	1.80	0.003	0.007
Propionate, mmol/L	27.4 ^b^	29.9 ^a^	30.6 ^a^	0.67	0.005	0.002
Butyrate, mmol/L	21.0	22.0	22.8	1.18	0.559	0.334
Acetate: propionate ratio	2.65	2.71	2.66	0.063	0.798	0.700

^a,b^ Means in the same row with different superscripts differ at *p* < 0.05. *p*-value is the observed significance level of the *F*-test for treatment; SEM, standard error of the mean. ^1^ Diets: Concentrate mixture in the control diet was replaced with *Chlorella vulgaris* microalgae (at 1%) and *Moringa oleifera* silage mixture at 0% (Control diet), 20% (MA20 diet) or 40% (MA40 diet), DM basis.

**Table 4:** Blood serum parameters of lactating Damascus goats fed diets containing *Moringa oleifera* and *Chlorella vulgaris* microalgae (*n* = 15).

	Diet ^1^				*p* Values	
	Control	MA20	MA40	SEM	Diet	Control vs. Others
Total proteins, g/dL	7.26	7.56	7.57	0.258	0.055	0.731
Albumin, g/dL	3.89	3.99	4.09	0.137	0.102	0.120
Globulin, g/L	3.37	3.56	3.49	0.069	0.160	0.079
Urea-N, mg/dL	39.8	39.6	40.4	1.62	0.552	0.052
Glucose, mg/dL	77.3 ^b^	85.4 ^a^	85.7 ^a^	0.45	<0.001	<0.001
Alanine aminotransferase, units/L	15.6	16.2	16.0	0.20	0.129	0.052
Aspartate transaminase, units/L	32.8	31.7	31.4	0.32	0.081	0.053
Triglycerides, mg/dL	164 ^b^	172 ^a^	171 ^a^	2.65	0.024	0.025
High-density lipoprotein, mg/dL	84.2 ^b^	94.3 ^a^	95.2 ^a^	0.60	<0.001	<0.001
Low-density lipoprotein, mg/dL	70.7	71.6	71.1	0.76	0.721	0.507
Antioxidant capacity, mg/dL	101 ^b^	110 ^a^	112 ^a^	2.40	0.003	0.008
β-Hydroxybutyrate, mg/dL	0.85	0.85	0.85	0.027	0.991	0.984
Nonesterified fatty acids, mg/dL	1.79	1.79	1.80	0.063	0.996	0.966

^a,b^ Means in the same row with different superscripts differ at *p* < 0.05. *p*-value is the observed significance level of the *F*-test for treatment; SEM, standard error of the mean. ^1^ Diets: Concentrate mixture in the control diet was replaced with *Chlorella vulgaris* microalgae (at 1%) and *Moringa oleifera* silage at 0% (Control diet), 20% (MA20 diet) or 40% (MA40 diet), DM basis.

**Table 5:** Milk yield and composition in lactating Damascus goats fed diets containing *Moringa oleifera* and *Chlorella vulgaris* microalgae (*n* = 15).

	Diet ^1^				*p* Values	
	Control	MA20	MA40	SEM	Diet	Control vs. Others
Production, g/d (unless stated otherwise)						
Milk	876 ^b^	1003 ^a^	1023 ^a^	32.0	0.005	0.001
Energy corrected milk (ECM)	839 ^b^	1066 ^a^	1030 ^a^	33.3	<0.001	<0.001
Fat corrected milk (4% FCM)	828 ^b^	1026 ^a^	998 ^a^	32.0	0.001	<0.001
Milk energy output, MJ/d	2.58 ^b^	3.29 ^a^	3.18 ^a^	0.103	<0.001	<0.001
Total solids	108 ^b^	137 ^a^	132 ^a^	4.3	<0.001	<0.001
Solids non-fat	76.3 ^b^	95.4 ^a^	92.0 ^a^	3.07	0.002	<0.001
Fat	31.8 ^b^	41.1 ^a^	39.8 ^a^	1.29	<0.001	<0.001
Protein	32.9 ^b^	40.7 ^a^	39.7 ^a^	1.41	0.006	0.002
Lactose	36.2 ^b^	46.2 ^a^	44.0 ^a^	1.40	<0.001	<0.001
Composition, g/kg unless stated otherwise						
Total solids	123	133	131	3.96	0.062	0.066
Solids non-fat	87.0	93.3	91.8	3.97	0.055	0.072
Fat	36.3 ^b^	40.2 ^a^	39.7 ^a^	0.37	<0.001	<0.001
Protein	37.6	39.8	39.5	2.52	0.091	0.205
Lactose	41.3 ^b^	45.2 ^a^	43.9 ^a^	0.58	0.001	<0.001
Milk energy content, MJ/kg	2.94 ^b^	3.22 ^a^	3.17 ^a^	0.022	<0.001	<0.001
Feed efficiency						
Milk: intake ratio	0.74 ^b^	0.87 ^a^	0.86 ^a^	0.028	0.004	0.009
ECM: intake ratio	0.71 ^b^	0.91 ^a^	0.89 ^a^	0.030	<0.001	<0.001
FCM: intake ratio	0.70 ^b^	0.88 ^a^	0.86 ^a^	0.028	<0.001	<0.001

^a,b^ Means in the same row with different superscripts differ at *p* < 0.05. *p*-value is the observed significance level of the *F*-test for treatment; SEM, standard error of the mean. ^1^ Diets: Concentrate mixture in the control diet was replaced with *Chlorella vulgaris* microalgae (at 1%) and *Moringa oleifera* silage at 0% (Control diet), 20% (MA20 diet) or 40% (MA40 diet), DM basis.

**Table 6:** Fatty acids profile (g/100 g total fatty acids) in milk of lactating Damascus goats fed diets containing *Moringa oleifera* and *Chlorella vulgaris* microalgae (*n* = 15).

	Diet ^1^				*p* Values	
	Control	MA20	MA40	SEM	Diet	Control vs. Others
C4:0	2.76	2.95	2.94	0.096	0.321	0.135
C6:0	2.07	2.10	2.16	0.043	0.322	0.273
C8:0	2.27	2.34	2.34	0.019	0.028	0.008
C10:0	5.05	5.13	5.15	0.042	0.179	0.069
C11:0	0.87	0.88	0.89	0.022	0.791	0.507
C12:0	3.16	3.21	3.17	0.028	0.429	0.387
C14:0	9.09	9.05	9.06	0.071	0.933	0.713
C14:1	0.68	0.68	0.69	0.005	0.169	0.094
C15:0	0.54 ^b^	0.53 ^b^	0.56 ^a^	0.006	0.022	0.310
C16:0	26.1 ^a^	24.9 ^b^	24.1 ^b^	0.20	<0.001	<0.001
C16:1	1.20 ^b^	1.23 ^b^	1.28 ^a^	0.013	0.002	0.003
C17:0	0.89	0.90	0.90	0.010	0.819	0.670
C18:0	16.5 ^a^	16.0 ^b^	16.3 ^a^	0.10	0.010	0.008
C18:1n9 *cis*	24.7 ^b^	25.5 ^a^	25.9 ^a^	0.21	0.001	0.005
C18:1n9 *trans*	2.42 ^b^	2.86 ^a^	2.84 ^a^	0.030	<0.001	<0.001
C18:2 *trans*-10, *cis*-12	0.27 ^b^	0.30 ^a^	0.31 ^a^	0.005	<0.001	<0.001
C18:2 *cis*-9, *trans*-11	0.18 ^c^	0.20 ^a^	0.19 ^b^	0.005	0.036	0.037
C18:3n3	0.17	0.18	0.18	0.005	0.225	0.086
C18:3n6	0.36 ^b^	0.39 ^a^	0.40 ^a^	0.006	0.001	<0.001
C20:0	0.66 ^a^	0.63 ^b^	0.63 ^b^	0.008	0.002	0.005
C20:5n3	0.15 ^b^	0.18 ^a^	0.17 ^a^	0.004	0.003	0.001
C22:5n3	0.19 ^b^	0.22 ^a^	0.21 ^a^	0.007	0.014	0.005
SFA	70.0	68.7	68.2	1.20	0.881	0.801
UFA	30.3	31.7	32.2	1.20	0.555	0.501
MUFA	29.0	30.3	30.7	1.22	0.617	0.538
PUFA	1.33 ^b^	1.46 ^a^	1.46 ^a^	0.014	<0.001	<0.001
Total CLA	0.45 ^b^	0.50 ^a^	0.50 ^a^	0.007	<0.001	<0.001
n6: n3 FA ratio	2.18	2.23	2.27	0.065	0.634	0.392
UFA: SFA ratio	0.43 ^b^	0.46 ^a^	0.47 ^a^	0.004	<0.001	<0.001
Athrogenicity index ^2^	2.17 ^a^	2.03 ^b^	1.98 ^b^	0.021	<0.001	<0.001

^a,b,c^ Means in the same row with different superscripts differ at *p* < 0.05. *p*-value is the observed significance level of the *F*-test for diet; SEM, standard error of the mean. CLA, conjugated linoleic acid (C18:2 *trans*-10, *cis*-12 and C18:2 *cis*-9, *trans*-11), MUFA, monounsaturated fatty acids; PUFA, poly unsaturated fatty acids; SFA, saturated fatty acids; UFA, unsaturated fatty acids. ^1^ Diets: Concentrate mixture in the control diet was replaced with *Chlorella vulgaris* microalgae (at 1%) and *Moringa oleifera* silage at 0% (Control diet), 20% (MA20 diet) or 40% (MA40 diet), DM basis. ^2^ Calculated according to Ulbricht and Southgate [25]: Atherogenicity index = (C12:0 + 4 × C14:0 + C16:0)/∑ of unsaturated fatty acids.

## Data Availability

Mean data are presented in the tables. The datasets generated during and/or analyzed during the current study are available from the corresponding author on reasonable request.
